# Spatial distances affect temporal prediction and interception

**DOI:** 10.1038/s41598-022-18789-2

**Published:** 2022-09-22

**Authors:** Anna Schroeger, Eric Grießbach, Markus Raab, Rouwen Cañal-Bruland

**Affiliations:** 1 Department for the Psychology of Human Movement and Sport, Institute of Sport Science, Friedrich Schiller University Jena, Jena, Germany; 2 Department of Psychology, Justus Liebig University Giessen, Giessen, Germany; 3 Department of Performance Psychology, Institute of Psychology, German Sport University Cologne, Cologne, Germany; 4 School of Applied Sciences, London South Bank University, London, UK

**Keywords:** Psychology, Human behaviour, Visual system

## Abstract

The more distant two consecutive stimuli are presented, the longer the temporal interstimulus interval (ISI) between their presentations is perceived (kappa effect). The present study aimed at testing whether the kappa effect not only affects perceptual estimates of time, but also motor action, more specifically, interception. In a first step, the original kappa paradigm was adapted to assess the effect in temporal prediction. Second, the task was further modified to an interception task, requiring participants to spatially and temporally predict and act. In two online experiments, a white circle was successively presented at three locations moving from left to right with constant spatial and temporal ISIs in between. Participants were asked to either (i) indicate the time of appearance of the predicted fourth stimulus (Exp. 1) or to (ii) intercept the predicted fourth location at the correct time (Exp. 2). In both experiments the temporal response depended on the spatial intervals. In line with the kappa effect, participants predicted the final stimulus to appear later (Exp. 1) or intercepted it later (Exp. 2), the more distant the stimuli were presented. Together, these results suggest that perceptual biases such as the kappa effect impact motor interception performance.

## Introduction

When we estimate the elapsed time between spatially separated and sequentially presented stimuli, our temporal judgments have been found to depend on the spatial distance between those stimuli. The more distant the stimuli are presented, the longer the temporal interval is perceived—a phenomenon referred to as the *kappa* effect^1,2^. Likewise, the influence of temporal intervals between the presentation of stimuli on judgments about their spatial distance is a well-known perceptual bias referred to as the *tau* effect^3,4^. However, whether the distorted perception of time and/or space also leads to biased motor responses remains an open question that we sought to address in the present study.

To start with, in the classical kappa and tau paradigms, the temporal and spatial biases were observed in judgment tasks in which a succession of three stimuli was visually presented and the interval between the first and second stimulus had to be compared to the interval between the second and third stimulus—either regarding their temporal duration or spatial length^1,3^. Later, modifications of this paradigm have been introduced extending the kappa and tau effects, for instance, to other sensory modalities (for instance, auditory perception^5,6^; tactile perception^7^) or tasks, including motor tasks^8,9^. Initial support for the transfer of these perceptual phenomena to motor performance was provided for both visual and auditory stimuli in a sequence learning task^8,9^. For instance, Sarrazin and colleagues made participants memorize a series of consecutively presented visual stimuli (i.e. dots) with varying spatial and temporal intervals between presentations^5,8^. In separate experiments, participants then had to reproduce either the spatial or the temporal configuration of the learned sequences motorically by either dragging and dropping visual markers to the memorized location (using a mouse) or pushing a button in the memorized rhythm. They found that in certain conditions, the reproduced temporal intervals were affected by their spatial extent (kappa effect) and vice versa (tau effect). These findings indicate that kappa and tau effects can be reproduced in memorized motor sequences, that is, a motoric reproduction of learned sequences. However, whether tau and kappa also affect the planning and execution of future actions such as in interception performance where the prediction of spatiotemporal trajectories of moving objects is crucial, remains yet to be determined.

In everyday tasks, temporal prediction is necessary to plan and execute future actions, such as when catching a ball or when avoiding collision with other objects (e.g., cars). A biased perception could hinder successful performance or, in the worst case, be disastrous, for instance, resulting in an accident. Whether kappa and tau effects not only influence perception, but also interception performance (i.e., action) remains to be examined. To address this lacuna, in the current study, we primarily aimed at systematically examining the impact of the kappa effect on interception performance. If the kappa- and tau-like effects found in memorizing and reproducing motor sequences transfer to prediction, we hypothesized that the kappa effect should not only show in a perceptual temporal estimation task, but that they should also impact motor interception performance.

**Figure 1:**
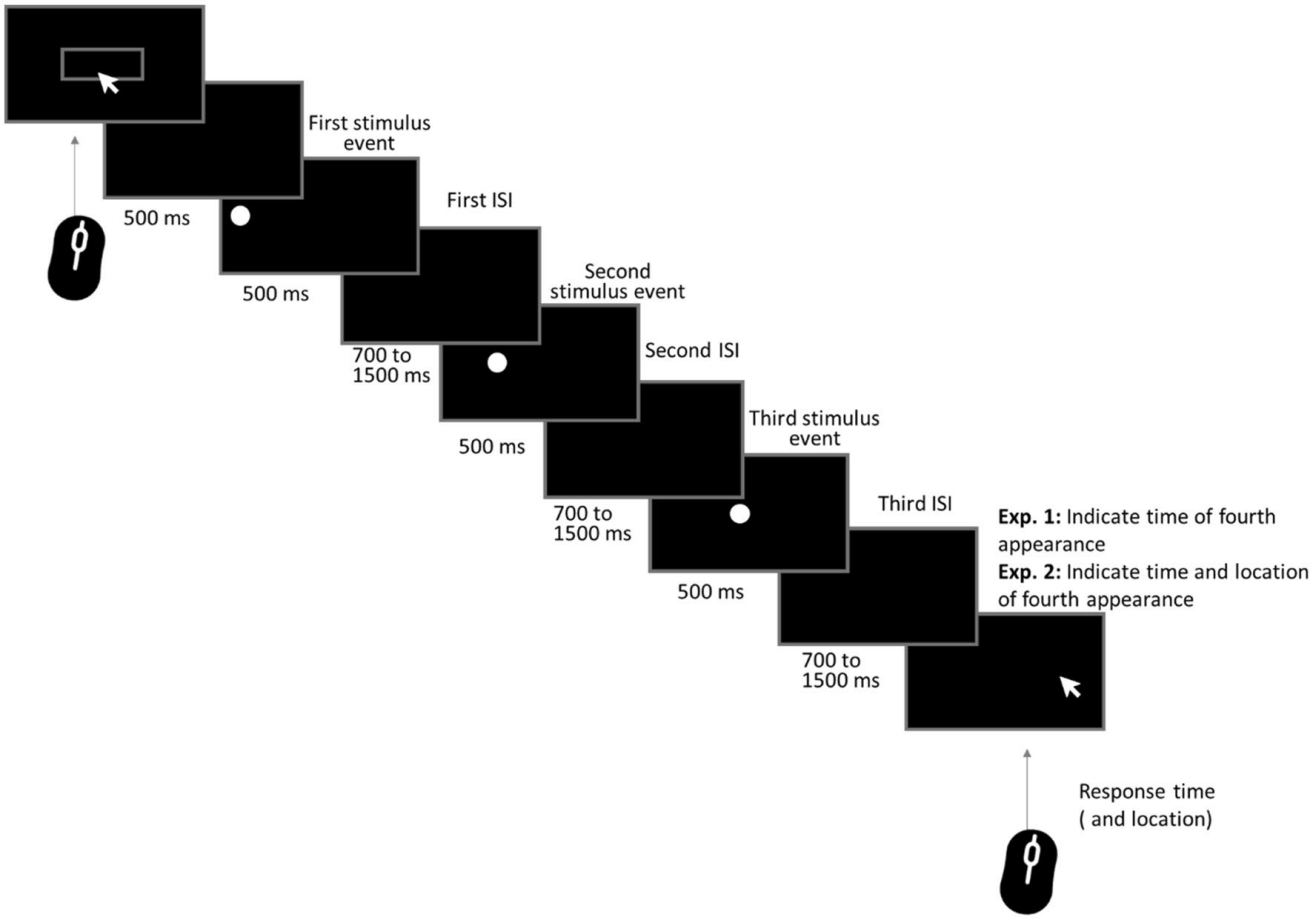
Structure of a single trial. Participants started each trial via a mouse/touchpad click. After a 500 ms pause, the visual stimulus was presented for 500 ms at the first location, it then disappeared for 700/900/1100/1300/1500 ms (interstimulus intervals = ISIs) before reappearing again for 500 ms at the second location (spatial intervals of 150/200/250/300/350 px). The disappearance and reappearance were repeated with the same temporal and spatial intervals and presentation times. After disappearing at the third location, participants were required to predict the fourth time (Exp. 1) and location (Exp. 2) of reappearance with the mouse or touchpad.

One problem with the classical paradigm typically used to investigate kappa is that it was not designed to test prediction, but to compare two previously experienced spatial or temporal intervals. In order to be able to assess whether the kappa effect modulates interception performance, we hence first had to modify the original paradigm and then to validate the modified paradigm. Therefore, in a first online experiment, the original kappa paradigm was adapted to assess the effect in a temporal prediction task. Participants were presented with a temporal succession of three spatially separated targets and were merely asked to provide a mouse click *when* they expected the next target to appear. After having validated that the modified paradigm produced kappa effects regarding the estimates of the appearance of the final stimulus, in a second online experiment the task was then further adapted to an interception task. More specifically, participants were asked to spatially and temporally intercept the target by predicting its next location and time of appearance. In contrast to previous studies, this latter interception task allowed us to measure a temporal and spatial response at the same time, or in other words, in a single move. In both tasks, spatial (150/200/250/300/350 px) and temporal (700/900/1100/1300/1500 ms) intervals were altered randomly between trials (see Fig. 1).

Previous research on spatiotemporal interrelations, suggests that sensory input might play an important role^10^. Due to high reliability of localization in the visual modality, but less precise timing^11,12^, the visual modality was suggested to be especially fruitful to assess effects of spatial features on timing. Additionally, research on such effects within the motor domain (as addressed in Exp. 2) is much needed. Based on previous work on the kappa effect and related studies considering spatiotemporal biases, we hypothesized that in both experiments, spatial manipulations would result in changes in the temporal response, indicating a kappa effect in both temporal prediction (Exp. 1) and interception (Exp. 2).

## Results

### Kappa effects in temporal prediction (Exp. 1) and interception (Exp. 2)

**Figure 2:**
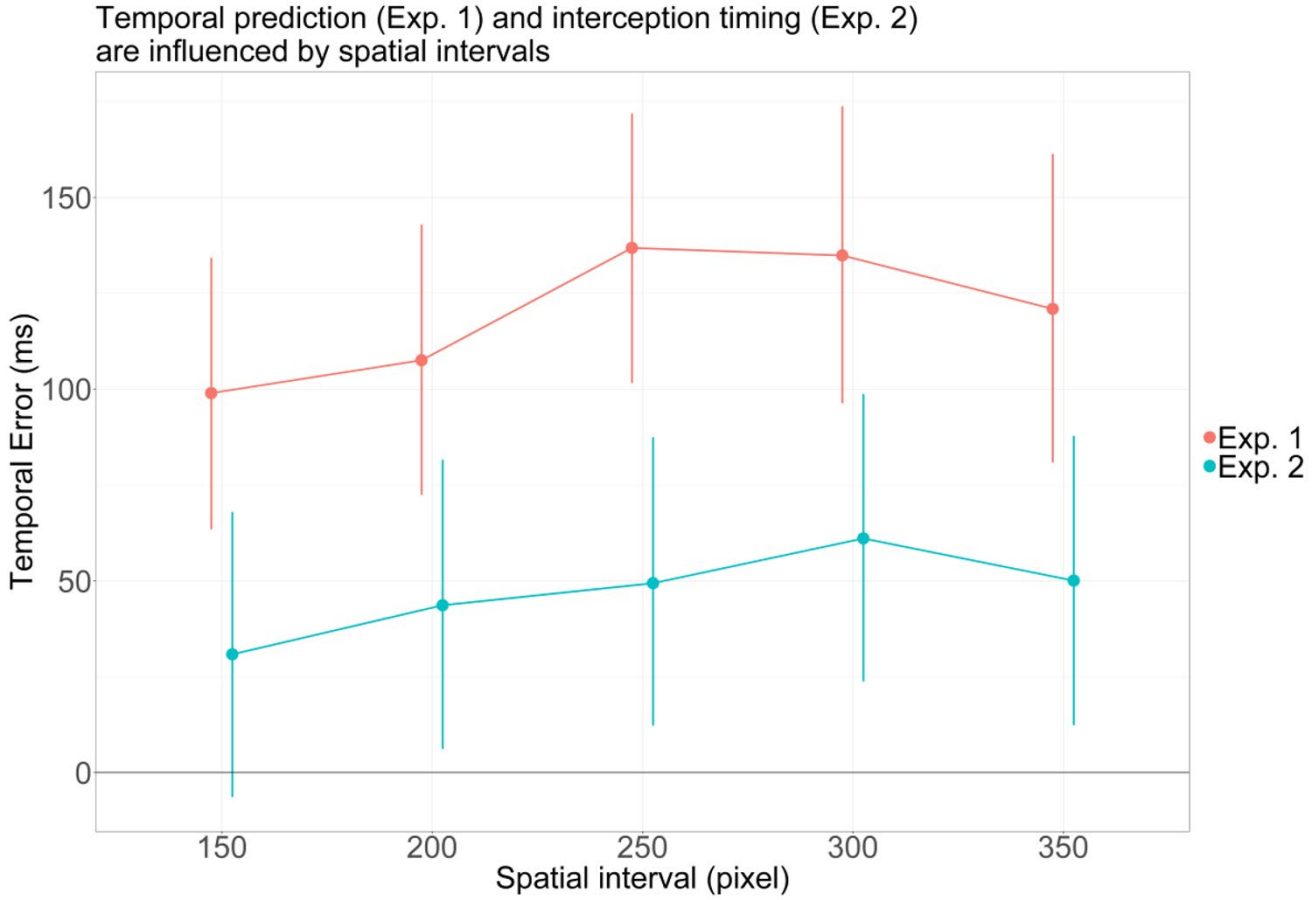
Model estimates (mean and 95% CrI) of the temporal error for the different spatial intervals for Exp. 1 and Exp. 2. Positive values indicate that the response was longer compared to the temporal ISI. Red: Results of Exp. 1 on temporal prediction: Participant’s response times slowed down for a distance between 150 to 250 pixels when the spatial distance increased (kappa effect). Blue: Results of Exp. 2 on interception timing: Participants reacted later when the distance increased (kappa effect), except for the 350 px interval.

In Exp. 1, overall participants tended to respond too late, that is, later than the fourth stimulus would have appeared, as indicated by a positive temporal error (β = 119.80, 95% CrI 85.08–154.86, P(β > 0) > 0.999). Most importantly, in line with the predictions of the kappa effect, the spatial distances between presentations influenced participants’ temporal response (see Fig. 2 and Table 1). More specifically, in the modified prediction paradigm of Exp. 1, participants predicted longer temporal interstimulus intervals (ISIs) between more distant presentations for the first three distances (see Table 1). The effect becomes compelling when comparing the intervals of 200 pixels with intervals of 250 pixels (29.34 ms, 95% CrI 10.91–47.74 ms, P(β > 0) = 0.999).

**Table 1:** Contrast estimates of the temporal error for consecutive spatial distances in Exp. 1 (temporal prediction). Positive values indicate that the response was longer in the consecutive level.

Effect	Estimate	95% credible interval	P (β > 0)
200 px vs. 150 px	8.56	[− 9.86 to 26.97]	0.82
250 px vs. 200 px	29.34	[10.91 to 47.74]	0.99
300 px vs. 250 px	− 1.99	[− 21.93 to 17.73]	0.42
350 px vs. 300 px	− 13.94	[− 32.93 to 4.91]	0.07

Similar to Exp. 1, participants tended to respond too late in Exp. 2, as indicated by a positive temporal error (β = 47.04, 95% CrI 12.38–82.01, P(β > 0) = 0.996). Most importantly, and as illustrated in Fig. 2 and Table 2, also in the interception paradigm the spatial intervals of the circle influenced participants’ temporal response. Again in line with a kappa effect, participants estimated the temporal delay between appearances of the circles to be larger with each consecutive spatial distance, except for the largest distance (see Table 2). The effect becomes compelling when comparing the intervals of 150 pixels with intervals of 300 pixels (30.24 ms, 95% CrI 7.62–52.85 ms, P(β > 0) = 0.996).

**Table 2:** Contrast estimates of the temporal error for consecutive spatial distances in Exp. 2 (interception). Positive values indicate that the response was longer in the consecutive level.

Effect	Estimate	95% credible interval	P(β > 0)
200 px vs. 150 px	12.80	[− 8.84 to 34.36]	0.88
250 px vs. 200 px	5.76	[− 16.01 to 27.64]	0.70
300 px vs. 250 px	11.68	[− 9.55 to 33.26]	0.86
350 px vs. 300 px	− 11.00	[− 32.91 to 11.04]	0.16

Except for these hypotheses-driven tests on the kappa effect, additional analyses regarding the effect of temporal ISI on the temporal response were run. As this was not the main concern of this manuscript, these additional exploratory analyses on the effect of temporal ISI, spatial distance, experiment version (1 vs. 2) and their interactions on the temporal response are reported in the supplementary material.

### Additional effect of temporal interstimulus intervals on response location in interception

**Figure 3:**
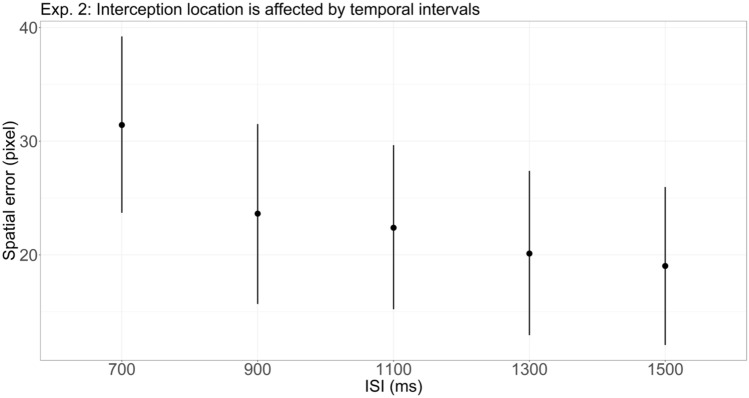
Model estimates (mean and 95% CrI) of the spatial error for different interstimulus intervals (ISIs) in Exp. 2. Positive values indicate that the response overshot the to-be-intercepted final location of the circle. Participant’s response location shifted toward the left side the longer the ISI.

Because the task in Exp. 2 allowed us to also examine the interception location, we further tested whether the temporal ISI impacted where participants intercepted, that is, whether there was a tau effect. Results showed that, overall, participants’ responses were spatially biased towards the right side of the actual stimulus location, indicating that they overshot the location (see Fig. 3 and Table 3). This is specified by a positive spatial error (β = 23.32 pixels, 95% CrI 16.42–30.22, P(β > 0) > 0.999). Notably, with increasing temporal ISI, the overshooting bias decreased.

**Table 3:** Contrast estimates of the interception location for consecutive temporal intervals. Positive values indicate that the response location was more shifted towards in the movement direction of the circle (overshooting) than for the previous temporal ISI level.

Effect	β (pixels)	95% CrI (pixels)	P(β > 0)
900 ms vs. 700 ms	− 7.80	[− 11.97 to − 3.63]	0.01
1100 ms vs. 900 ms	− 1.24	[− 5.66 to 3.13]	0.29
1300 ms vs. 1100 ms	− 2.27	[− 6.43 to 1.85]	0.14
1500 ms vs. 1300 ms	− 1.09	[− 5.33 to 3.18]	0.30

## Discussion

The purpose of the present study was to test whether the well-established perceptual kappa effect also impacts interception performance. In a first experiment, the traditional kappa design was adapted to a temporal prediction task. In a second experiment, additional modifications of the task allowed to assess the kappa effect in motor interception. In line with the kappa effect, participants’ temporal prediction increased with increasing distance between stimuli in Exp. 1. Similarly, in Exp. 2 the timing of interception was affected by the distance between stimuli. Specifically, participants intercepted the target stimulus later when distances between stimuli increased (kappa effect)^1,2,13^, with an exception for the largest spatial interval (350 px).

Importantly, there is some evidence that the temporal ISI moderated the kappa effect in Exp. 2. For some comparisons the effects seem to diminish or even reverse (see Supplementary Fig. S1). Additionally, there was an overall trend in the interception task (Exp. 2) that participants temporally overshot short and undershot long ISIs, potentially indicating a tendency towards the center. This might reflect an overall increased uncertainty in the interception paradigm compared to the temporal prediction task, in which the temporal intervals were generally overshot but lesser so for longer temporal ISIs. We neither had a priori hypotheses concerning the outcome of these additional, exploratory analyses nor are we aware of evidence providing robust support for these initial empirical findings. It follows that future research is needed to examine the potential impact of temporal ISIs on the kappa effect.

Together, the effects found in both experiments are in line with previous research on the kappa effect showing that temporal intervals between a sequence of stimuli are judged to have a longer duration when the stimuli are more distant^1,2,13,14^. Therefore, our findings extend earlier research by showing that the kappa effect transfers to motor actions. More specifically, adding to earlier reported effects on motor sequence learning^8,9^, the current findings reveal an impact of kappa effects—and hence spatiotemporal biases—on temporal prediction and motor interception performance. The findings also enrich current debates about the coupling of perception and action^15–17^ and the impact of illusions, in particular, visual illusions such as the Müller-Lyer and Ebbinghaus illusions on motor performance for which some studies reported no evidence^18,19^, positive evidence^20–22^ and even mixed evidence^23^.

When comparing the size of the temporal errors between Exp. 1 and Exp. 2 (see Fig. 2), it becomes apparent that the size of temporal errors in the mere prediction task was almost twice as large as the temporal errors in the interception task. This may be at least partially explained by previous research on time to contact estimations showing that a purely temporal response towards motion objects (similar to Exp. 1) does not exclusively depend on temporal, but also speed information^24^. If true, then it is reasonable to assume that participants may have used and perhaps integrated velocity, timing and spatial cues to perform the interception task in Exp. 2. In addition, the interceptive movement itself and/or its effects (i.e. the cursor moving across the screen) are likely to have provided additional online feedback allowing to update the interceptive movement, thereby contributing to smaller temporal errors.

Importantly, the additional analyses reported in the supplement indicate that the temporal error was nearly identical between experiments for the shorter temporal ISIs. With increasing time between stimulus presentations, the temporal error was then reduced, more so in the interception task (Exp. 2) which even results in undershooting. As indicated above, this finding might be interpreted as an overall tendency to the center (reacting later for short and earlier for long temporal ISIs) which could reflect higher uncertainty in the interception task.

Overall, the finding that participants reacted too late contrasts with studies on synchronizing actions with events^25^ or reproduction of rhythms^26^. This highlights the different demands of such tasks. When participants perform an action repeatedly and try to temporally synchronize it with a stimulus signal, the action performance tends to precede the stimulus event. This finding is interpreted as supporting the Paillard-Fraisse hypothesis^27^ which states that temporal events are temporally ordered according to the temporal succession of their representational codes in the brain. Due to longer processing times for distal events (e.g., sensory information from hand to brain) compared to fast processing of auditory or even visual stimuli, actions must be executed in advance to temporally synchronize both codes. However, this preceded timing is typically established after a few repeated taps, which are not typically included in the analyses of the asynchrony. As the current task only allowed one tap per trial, no sensory feedback for following taps within a trial of the same temporal ISI was available. In general, the current task does not allow to test for brain-code coincidence as for the event in which the participant clicks, no stimulus event is presented. This might explain why we did not find participants to react early in the current task.

Importantly, the results should be discussed in the context of the framework on representational (or ‘explicit’) vs. emergent (or ‘implicit’) timing^28^ which states that different timing processes can be dissociated across various tasks^29,30^. Representational timing refers to an explicit representation of a temporal goal and was found to be prevalent in movement initiation, whereas implicit timing was shown for movement duration where timing emerges through the control of other kinetic factors such as movement speed^28,31^. In implicit timing tasks, timing can be seen as a result of controlling movements, without the explicit goal of reaching a point in time in mind^28^.

Here we used the wording ‘temporal representation’ suggesting that explicit timing was addressed. And indeed, when comparing the current task to those of previous experiments, more similarities between explicit timing and interception/temporal prediction than for implicit timing can be identified: Temporal prediction and interception with a mouse (touchpad) both require movement initiation (similar to tapping or intermittent circle drawing) instead of continuous movements as in the implicit continuous circle drawing task. Additionally, the concrete temporal intervals directly relate to the pauses implied in tapping or even intermittent circle drawing both representing explicit timing. If true, the action-based kappa effect, as assessed in the current study, might rely on explicit representation of timing meaning that the presented results do not necessarily transfer to implicit timing tasks. This is especially important, given that other interception tasks such as catching a ball have been suggested to be driven by implicit time encoding^28^. For instance, a goalkeeper catching a ball might translate his main goal of reaching a certain location in time into subgoals, like increasing movement velocity. This subgoal might be actively controlled to implicitly achieve the timing goal. Importantly, time encoding might even have differed between the two experiments: Similarities to the explicit tapping task are especially evident for Exp. 1 on temporal prediction. In contrast, one might argue that Exp. 2 which assessed manual interception might have triggered implicit timing by, for instance, controlling movement velocity. If true this would suggest that kappa affects both components, explicit representations, and implicit timing (or related components of movement control). Nevertheless, this conclusion is only speculative and a profound evaluation on the paradigm and the implied temporal processes is needed.

Another finding of the interception task was that with increasing temporal ISIs participants overshot the target location less, which may be interpreted as a reversed tau effect, and therefore contrasts with the previously reported perceptual tau effects^3,4^. While an inverted kappa effect has already been reported for auditory stimuli^32^, to our knowledge, this is the first time, an inverted tau effect was found. However, given that for several localization biases also inverted effects (i.e. biases in the opposite direction) have been reported, it might not be surprising to find such an inversion also for the tau effect. For instance, in contrast with the Representational Momentum effect, typically showing that a target’s movement offset location is overshot^33,34^, researchers have repeatedly reported an opposite effect, called the offset-repulsion effect^35,36^. Similarly, seemingly contradictory findings have been reported for movement onset locations described as the Fröhlich effect^37^—that is, the perceived onset location of stimuli in motion is shifted in motion direction—or its’ inversion, the onset-repulsion effect^38^. The original kappa and tau effects (but not their inversions), are often explained by models assuming that expectations about an underlying motion with constant velocity between presentations (slow speed priors) account for the biases^39,40^. A novel theoretical account, referred to as the speed prior hypothesis^41,42^, which is also based on prior speed expectations likewise predicts and explains the reversed findings for several biases. This includes the aforementioned offset and onset repulsion effects, but also the inversed versions of kappa and tau effects. In specific, similar to the slow speed hypothesis, this hypothesis predicts smaller/larger spatial and shorter/longer temporal intervals depending on participants’ *expectations* about the speed (priors), which may be different from the actual speed. Most importantly, it also accounts for possible inversions of the effects, depending on the velocity range administered in the task (i.e., the combination of temporal and spatial intervals). For slower presented speeds, a positive relationship between speed and the amount of overshooting is expected (length extension), while as soon as reaching a certain speed (half the speed of the prior), the overshooting should be reduced with increasing speed and even result in undershooting when exceeding the prior speed^41,42^. It is conceivable that the chosen temporal and spatial intervals in the current study perhaps met the reversal point for the kappa effects, therefore first resulting in a positive effect and then, for longer spatial intervals (where the speed exceeded half of the prior speed) an inversion of this relationship. In addition, the speed prior hypothesis^41,42^ may also explain the inverted tau effect: If the chosen spatial and temporal intervals resulted in a ‘medium’ speed range (i.e., speeds between half of the prior speed and the prior speed), this should have resulted in the observed inversed tau effect.

Finally, next to the many advantages of online studies, like access to a larger and more diverse sample, more efficient/economic use of resources, and reduction or even elimination of experimenter effects^43^, they also have a few limitations such as no or less control over participants’ behavior during experimentation, used screen sizes, the distance between participants and their screens and the fact whether they finally used a mouse or touchpad for performing the interception task. In Exp. 1 and Exp. 2, 24 out of 57 and 32 out of 53 respectively, participants reported to have used a computer mouse. Concerning the control of participants’ behavior, for instance, few participants additionally reported that they produced rhythmical sounds with their mouth to support their performance in the temporal task. However, despite these challenges and potential limitations, we deem it unlikely that such behaviors account for our results and findings because we not only found the predicted kappa effects, but we also replicated it across two separate online experiments. Comparisons of online and lab-based studies, so far revealed similar results, emphasizing the validity of web experiments in cognitive and perceptual research^44^. Regardless, we call for more research examining spatiotemporal biases in interception performance that allows for better controlled and ecologically more valid motor responses such as interceptive movements in a Virtual Reality setting.

## Methods (Exp. 1 and Exp. 2)

### Participants

Previous research has reported effects for sample sizes of n = 6 to n = 12^8,45^. Given that the current study administered a predictive (and motor) response instead of verbal comparisons (as opposed to previous studies^1^), and was run with limitations regarding the control of potentially relevant factors (e.g., screen size), a sample size of approximately 55 was intended to compensate for higher noise (e.g., motor noise, less controlled environment). In Experiment 1, data of 57 participants who took part in the online experiment were further processed (age: mean = 25.1 years, min = 18 years, max = 48 years; Handedness: 52 right-handed, 4 left-handed, 1 no preference; gender: 40 females, 17 males). 32 additionally recruited participants had to be excluded from further analysis, because they either did not finish at least the first block of 25 trials (n = 15), did not follow the instruction (n = 15), were too young (n = 1), or erroneously took part in both Experiments (n = 2). Whether participants followed the instruction to ignore the spatial position of the ball was indicated by a significant effect of distance between stimuli on participants’ response location. In Experiment 2, 53 newly recruited participants were included in the analyses (age: mean = 25.6 years, min = 19 years, max = 55 years; Handedness: 44 right-handed, 9 left-handed; gender: 32 females, 20 males, 1 diverse). An additional 48 participants were recruited but excluded because they did not finish more than a few trials (< 25 trials, n = 41), or did not follow the instruction (n = 7). To control whether participants followed the instructions to predict the circle spatially and temporally in Exp. 2, we checked whether the temporal ISI predicted the response time and whether the circle jumping distance predicted participants’ response location for each individual.

In both experiments, participants provided informed consent prior to participation. A link to the online study was distributed via mailing lists at national universities and through communication with students at the local sports science institute. The study was approved by the local ethics committee (Ethical Commission of the Faculty of Social and Behavioural Sciences at the Friedrich Schiller University Jena, number of approval: FSV 21/033). We confirm that all research was performed in accordance with the Declaration of Helsinki.

## Materials

Both experiments were created with OpenSesame v3.3.4^46^ using OSWeb v1.3.13. We used Jatos v3.6.1^47^ as backend software for server-related management. During each trial, a white circle (20 pixels) was presented on a black background. The circle first appeared at − 600 pixels from the center of the screen (negative values are to the left of the center, positive values to the right). Afterwards, the circle dis- and reappeared two times one after another moving to the right with spatial intervals of 150/200/250/300/350 pixels. Therefore, the correct extrapolated positions for the third event were − 150/0/150/300/450 pixels from the center of the screen. The spatial intervals were chosen to resemble a relatively wide range of stimuli within the boundaries set by common screen dimensions (1920 × 1280 px). At each location, the circle was presented for 500 ms and the temporal ISIs between presentations were 700/900/1100/1300/1500 ms. The presentation times and intervals are within the range of previously used times^1,14^ and should allow for accurate timing with common refresh rates of screens (e.g., 60 Hz).

Participants were instructed to indicate via mouse/touchpad click when (Exp. 1) or when and where (Exp. 2) they expected the stimulus to appear for the fourth time. That means that in Exp. 1 participants had to perform a temporal prediction task, whereas in Exp. 2 they were expected to intercept the target (i.e. the final stimulus).

### Procedure

Before the experiment started, participants provided informed consent and filled out demographic questions regarding handedness, age, sex, etc. Participants received verbal instructions supported by a visual depiction.

Figure 1 displays the structure of a trial. To center the mouse position at the start of a trial, participants had to click a start button in the center of the screen. Participants’ task was to watch the succession of three visual stimuli (circles) presented with constant temporal and spatial intervals in between and then predict (Exp.1) or intercept (Exp. 2) the fourth (location and) time of appearance. The temporal ISIs (5 levels) and distances (5 levels) varied randomly between trials in one block, resulting in 25 trials per block. The whole experiment included 5 blocks (repetitions), resulting in a total of 125 trials. The duration of the experiment was roughly 20 min, which we thought would be a reasonable amount of time for an online study.

### Data analysis

We used R^48^ version 4.1.2 for statistical analysis. The whole data set consisted of 6361 trials from 57 participants in Exp. 1, and 6239 experimental trials from 53 participants in Exp. 2.

Because participants might have reacted erroneously to the wrong stimulus presentation (reaction towards earlier presentation or overseen presentation), outliers defined as extreme values more than 3 times the interquartile range from the 25% or 75% quantile were excluded for each participant. This led to an exclusion of 50 and 40 trials in Exp. 1 and 2, respectively. After exclusion, the statistical analysis included 6311/6361 (99.21%) from 57 participants in Exp. 1 and 6199/6239 (99.35%) of all trials from 53 participants in Exp. 2.

Our first aim was to analyze the influence of the spatial distance between stimuli on response timing (kappa effect). These analyses included repeated measures on the level of subjects which could correlate. To allow for correlation within subjects we opted to use a Mixed Model approach^49^. Additionally, we opted for a Bayesian approach because of more robust analysis when fitting mixed models and to avoid convergence problems^50^.

Model fitting was done with the brms package^51^ which provides an interface to fit Bayesian models using Stan^52^. We mostly followed the workflow and recommendation of Kruschke^53^. This includes prior predictive checks to choose sensible priors, converging checks of the sampling method of the posterior distribution of model parameters, and posterior predictive checks to get a (rough) sense of whether the model fitted the data adequately. Our reproducible analyses and data can be found at https://doi.org/10.17605/OSF.IO/675J4. In the Linear Mixed Model, the fixed effect spatial distance (factor with 5 levels, 150–350 pixels) was included with a sliding contrast, comparing consecutive levels. Additionally, to estimate the variance and allow for correlations between measures, we included a random intercept and a random slope for participants. We used weakly informative priors, which are defined by a broad (not flat) distribution of priors to mitigate the influence of unrealistic parameter values like a 100 s temporal error. Weakly informative priors are recommended compared to uninformative (flat) priors, to avoid overfitting by constraining the solution space of parameter values. Data from a yet unpublished study served as an estimation for the prior distributions. Our second aim was to analyze the influence of temporal ISI on response location. We ran the same analysis but with temporal ISIs (factor with 5 levels, 700–1500 ms) as a predictor for the spatial error.

The Bayesian Model provides a posterior distribution for every model parameter, representing the certainty of where the parameter lies in a specific range. To communicate this (un)certainty, we summarized the posterior distribution and present the estimated mean, the 95% credible interval, and the probability that the parameter is larger than 0.

## Supplementary Information

Supplementary Information.

## Data Availability

The data and materials for all experiments are available at https://doi.org/10.17605/OSF.IO/675J4. For further information please contact Anna Schroeger (annaschroeger@gmail.com).
