# Supplementary material for: Spatial distances affect temporal prediction and interception
Source: Sci Rep. 2022 Sep 22;12:15786. doi: 10.1038/s41598-022-18789-2 (PMC9499971; doi:10.1038/s41598-022-18789-2)
Supplement: Supplementary file 1 — Supplementary Information. [file 41598_2022_18789_MOESM1_ESM.docx]

Spatial distances affect temporal prediction and interception

Anna Schroeger^1,2^, Eric Grießbach^1^, Markus Raab^3,4^, Rouwen Cañal-Bruland^1^

1 Department for the Psychology of Human Movement and Sport, Institute of Sport Science, Friedrich Schiller University Jena, Germany

2 Department of Psychology, Justus Liebig University Giessen, Germany

3 Department of Performance Psychology, Institute of Psychology, German Sport University Cologne, Germany

4 School of Applied Sciences, London South Bank University, UK

Author Note

The project was funded by the Deutsche Forschungsgemeinschaft (DFG), project number: CA 635/2-2 awarded to Rouwen Cañal-Bruland and RA 940/15-2 awarded to Markus Raab

# Supplement

## Methods

The distribution link contained a request that participants should do the experiment on a PC or Laptop with a mouse or touchpad.

In Exp. 1, 15 participants were excluded because they did not follow the instruction to ignore the spatial position of the ball. This was indicated by a significant effect of distance between stimuli on participants’ response location. Two participants were excluded because they took part in both experiments and one participant was too young.

To control whether participants followed the instructions to predict the circle spatially and temporally in Exp. 2, we checked whether the temporal ISI predicted the response time and whether the circle jumping distance predicted participants response location for every individual. To do so, we ran a linear model with the predictor subject and the interaction between subject and ISI predicting the outcome response timing. 7 participants did not show a significant ISI effect, indicating that they did not follow the timing instruction and leading to the exclusion of these participants. We did the same analysis for the spatial distance, for which every participant had a significant effect, indicating that they followed the spatial instruction.

## Exploratory analysis of the temporal error

While our main analysis only focused on the kappa effect (main effect of spatial interval on the temporal error), we also exploratively analyzed the other factors we manipulated in the experiment and all interactions: ISI and instructions between Exp. 1 and 2. We fitted ISI as a slope centered at zero scaled to numerical values between -2 to 2. This makes interpretation easier (every 200 ms “level” increases temporal error by the estimated time in ms). Experiment was added as a factor with two levels (Exp. 1 and Exp. 2) with a sum contrast.
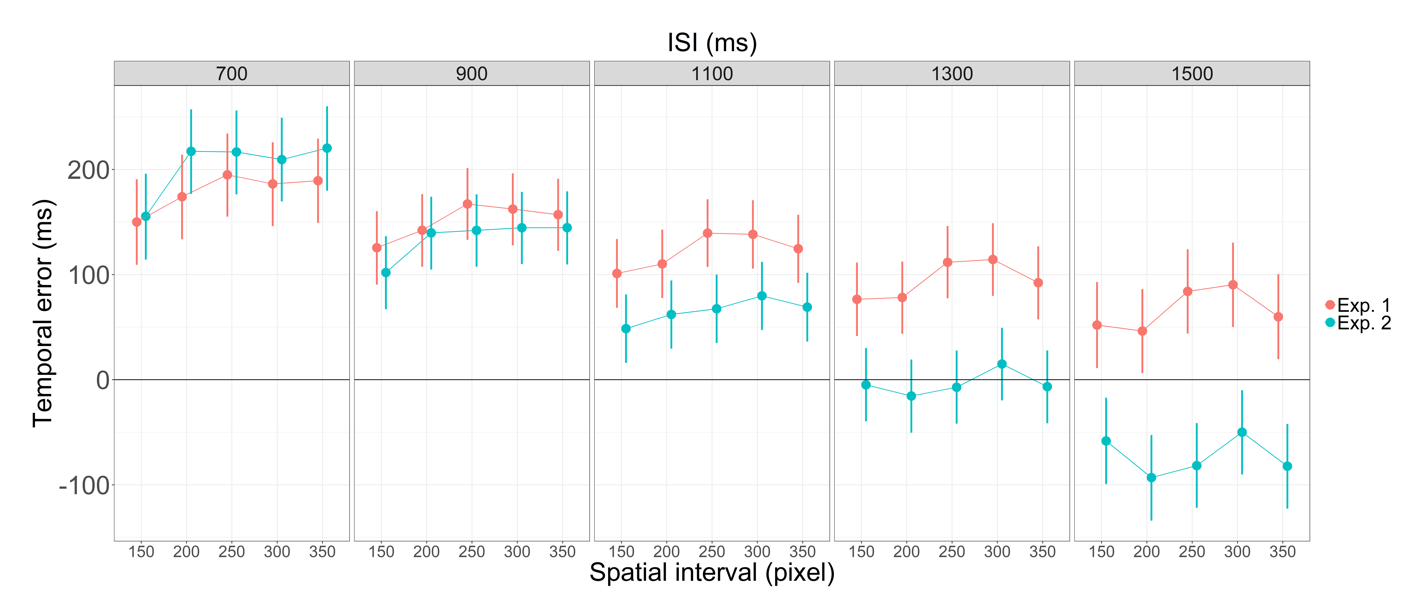


**Figure S1. Interaction between ISI, Experiment, and spatial Interval on the temporal error.** Note that the difference in the first two levels of spatial intervals decreases with ISI in Exp. 2.

From this explorative analysis of the interaction between spatial interval, ISI, and Experiment a few noticeable findings may be examined in future experiments. First, there is some evidence that the kappa effect is moderated by ISI timing, but only in Exp. 2 (see Figure S1). More specifically, the kappa effect between the first two levels of the spatial interval becomes more unlikely for higher ISIs (β= -16.73, 95% CrI = -36.18 to 2.60, P(β>0) = 0.04, see Figure S1). For that reason, the difference in the kappa effect is especially striking between the fastest and slowest ISI. For 700 ms ISI there is a clear kappa effect between the first to the second spatial interval in Exp. 2 (61.75 ms, 95% CrI = 26.87 to 95.49 ms). However, this effect is completely missing and even negative for the 1500 ms ISI (-34.82, 95% CrI = -0.45 to -69.71).


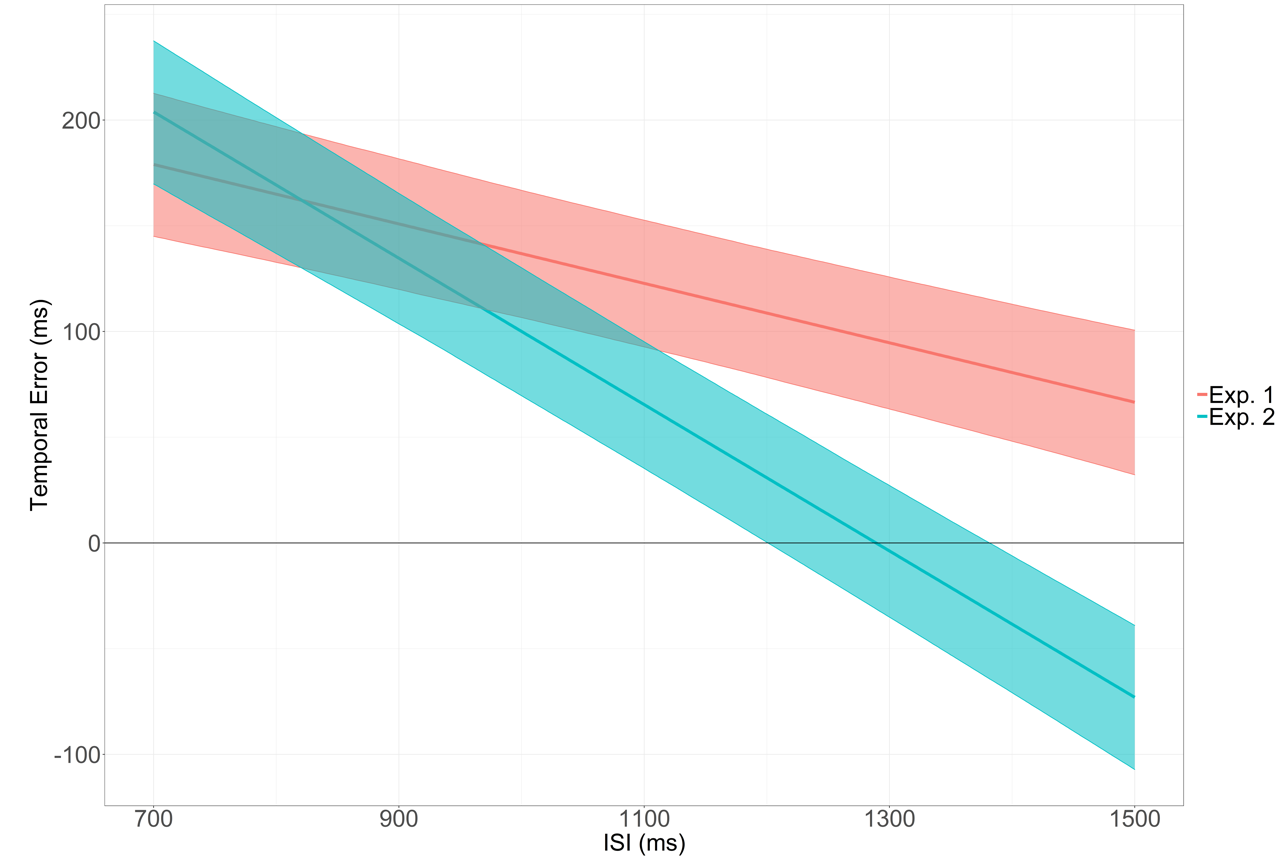


| **Figure S2. Differences in the influence of ISIs on the temporal error in Exp. 1 and Exp. 2.** Positive values indicate that the response was longer compared to the temporal ISI. The temporal error (response time) decreased with longer ISIs in both experiments. This decrease in response time was higher in Exp. 2. |
| --- |

Second, the temporal error (or the relative response time) decreased with temporal ISI (β= -48.67, 95% CrI = -56.11 to -41.30, P(β>0) < 0.01, see Figure S2) and future research may test alternative explanations for such a finding. More importantly, this decrease was amplified in Exp. 2, where participants had an interception task (β= -41.11, 95% CrI = -48.03 to -34.23, P(β>0) < 0.01, see Figure S1 and Figure S2). All main effects and interactions are reported in Table S1.

**Table S1. Estimates of the full model with spatial interval, ISI and experiment predicting temporal errors.** Each parameter is summarized as the mean odds ratio, the 95% CrI, and the probability that the posterior is smaller than one. Parameters with a high probability of being smaller or greater than one are highlighted with a bold font (< 0.05 or > 0.95). For contrasts, see methods and the description above.

| **Effect** | **Overall** | | | | | **Difference Exp. 2 vs. Exp. 1** | | | | |
| --- | --- | --- | --- | --- | --- | --- | --- | --- | --- | --- |
|  | **β** | **95% credible interval** | | | **P(β>0)** | **β** | **95% credible interval** | | | **P(β>0)** |
| Intercept | 94.10 | [64.34 | to | 123.59] | 0.99 | **-57.34** | **[-67.61** | **to** | **-47.11]** | **0.01** |
| 200 px vs. 150 px | 11.32 | [-2.60 | to | 25.22] | 0.95 | 4.33 | [-22.80 | to | 31.31] | 0.62 |
| **250 px vs. 200 px** | **17.31** | **[3.42** | **to** | **31.19]** | **0.99** | **-23.88** | **[-50.92** | **to** | **3.52]** | **0.04** |
| 300 px vs. 250 px | 5.59 | [-8.25 | to | 19.49] | 0.79 | 13.40 | [-13.55 | to | 40.40] | 0.84 |
| **350 px vs. 300 px** | **-12.21** | **[-26.45** | **to** | **2.07]** | **0.05** | 2.99 | [-23.85 | to | 30.11] | 0.59 |
| **Timing** | **-48.67** | **[-56.11** | **to** | **-41.30]** | **0.01** | **-41.11** | **[-48.03** | **to** | **-34.23]** | **0.01** |
| **200 px vs. 150 px: ISI** | **-15.80** | **[-26.02** | **to** | **-5.54]** | **0.01** | **-16.73** | **[-36.18** | **to** | **2.60]** | **0.04** |
| 250 px vs. 200 px: ISI | 3.61 | [-6.02 | to | 13.39] | 0.77 | -1.22 | [-20.28 | to | 17.81] | 0.45 |
| 300 px vs. 250 px: ISI | 6.76 | [-2.84 | to | 16.26] | 0.92 | 6.03 | [-13.16 | to | 25.28] | 0.73 |
| **350 px vs. 300 px: ISI** | **-9.59** | **[-19.40** | **to** | **0.22]** | **0.03** | -2.45 | [-21.54 | to | 16.71] | 0.40 |
